# Cost-effectiveness analysis of cinacalcet vs. paricalcitol in the treatment of hyperparathyroidism secondary to chronic kidney disease

**DOI:** 10.1590/2175-8239-JBN-2022-0126en

**Published:** 2023-04-03

**Authors:** Daniela Ponce, Marilia Mastrocolla de Almeida Cardoso, Juliana Rodrigues Machado Rúgolo, Silvana Andrea Molina, Luis Gustavo Modelli de Andrade, Daniel da Silva Pereira Curado

**Affiliations:** 1 Universidade Estadual Paulista Júlio de Mesquita Filho, Faculdade de Medicina, Hospital das Clínicas, Núcleo de Avaliação de Tecnologia em Saúde, Botucatu, SP, Brazil.; 2 Ministério da Saúde, Departamento de Gestão e Incorporação de Tecnologias e Inovação em Saúde, Brasília, DF, Brazil.

**Keywords:** Hyperparathyroidism, Secondary, Renal Insufficiency, Chronic, Cinacalcet, Paricalcitol, Cost-Effectiveness Evaluation, Hiperparatireoidismo Secundário, Insuficiência Renal Crônica, Cinacalcete, Paricalcitol, Avaliação de Custo-Efetividade

## Abstract

**Introduction:**

For the reduction of PTH levels, two classes of drugs are available in the Brazilian market: non-selective and selective vitamin D receptor activators and calcimimetics. Among the mentioned drugs, the SUS provides oral calcitriol, paricalcitol and cinacalcet.

**Objectives:**

Develop cost-effectiveness (CE) and budgetary impact (BI) analysis of cinacalcet versus paricalcitol for patients on dialysis with SHPT, from the perspective of SUS.

**Methodology:**

A decision tree model was constructed for CE analysis, which considered the outcome of avoided parathyroidectomy and a time horizon of 1 year. As for the BI analysis, two scenarios were considered, one of which was measured demand and other epidemiological, based on data from the Brazilian Society of Nephrology (BSN).

**Results:**

The CE analysis showed that the use of cinacalcet results in one-off savings of R$1,394.64 per year and an incremental effectiveness of 0.08, in relation to avoided parathyroidectomy. The incremental CE ratio (ICER) was - R$ 17,653.67 per avoided parathyroidectomy for cinacalcet, as it was more effective and cheaper compared to paricalcitol. As for the BI analysis, it was estimated that the incremental BI with the expansion of the use of cinacalcet in the SUS will be between - R$ 1,640,864.62 and R$ 166,368.50 in the first year, considering the main and the epidemiological scenarios. At the end of 5 years after the expansion of use, an BI was estimated between - R$ 10,740,743.86 and - R$ 1,191,339.37; considering the same scenarios.

**Conclusion:**

Cinacalcet was dominant to avoid parathyroidectomies, being cost-effective.

## RESUMO

**Introdução:**

Para a redução dos níveis do paratormônio (PTH) estão disponíveis no mercado brasileiro duas classes de medicamentos: ativadores do receptor da vitamina D (não seletivos e seletivos) e calcimiméticos. Dentre os medicamentos supracitados, o SUS disponibiliza calcitriol oral, paricalcitol e cinacalcete.

**Objetivos:**

Desenvolver análise de custo-efetividade (CE) e de impacto orçamentário (IO) do cinacalcete versus paricalcitol para pacientes em diálise com HPTS, na perspectiva do SUS.

**Metodologia:**

Foi construído um modelo de árvore de decisão para a análise de CE, que considerou o desfecho paratireoidectomia evitada e um horizonte temporal de 1 ano. Quanto à análise de IO, foram considerados dois cenários, um de demanda aferida e outro de abordagem epidemiológica, baseado nos dados da Sociedade Brasileira de Nefrologia (SBN).

**Resultados:**

A análise de CE mostrou que o uso de cinacalcete resulta em economia de R$ 1.394,64 ao ano e efetividade incremental de 0,08, em relação a paratireoidectomia evitada. A razão de CE incremental (RCEI) foi de - R$ 17.653,67 por paratireoidectomia evitada para o cinacalcete, já que se mostrou mais efetivo e mais barato comparado ao paricalcitol. Estimou-se que o IO incremental com a ampliação do uso do cinacalcete no SUS estará entre - R$ 1.640.864,62 e R$ 166.368,50 no primeiro ano, considerando os cenários principal e epidemiológico baseado nos dados da SBN. Já ao final de 5 anos após a ampliação do uso, estimou-se um impacto incremental entre - R$ 10.740.743,86 e - R$ 1.191.339,37; considerando os mesmos cenários.

**Conclusão:**

Cinacalcete foi dominante para evitar paratireoidectomias, sendo custo-efetivo.

## Introduction

Hyperparathyroidism secondary (SHPT) to chronic kidney disease (CKD) is characterized by elevated serum levels of parathyroid hormone (PTH), hyperplasia of the parathyroid glands, high turnover bone disease and cardiovascular disease^1, 2, 3^. The PTH level considered adequate for patients with CKD stage 5D is situated between two and nine times the threshold value of the dosage method^1^. According to the census of the Brazilian Society of Nephrology (SBN), in 2020, it is estimated that 144,779 patients are undergoing dialysis treatment in Brazil^4^. Of these, approximately 18% had PTH levels above 600 pg/mL in 2019; while in 2014 they were around 26%, suggesting that there was some impact in reducing PTH levels with the incorporation of paricalcitol and cinacalcet and implementation of PCDT in 2017. For the reduction of PTH levels, three classes of drugs are available on the Brazilian market: non-selective vitamin D receptor activators (calcitriol and alfacalcidol), selective VDR activators (paricalcitol) and calcimimetics (cinacalcet)^5^. Among the aforementioned drugs, SUS makes oral calcitriol available, with its intravenous presentation being discontinued in 2020, and oral alfacalcidol in 2017. Regarding paricalcitol, its availability in SUS is aimed at patients with PTH equal to or greater than 500 pg/mL and, for cinacalcet, for patients with PTH levels above 800 pg/mL, which may be the first option in the presence of hypercalcemia and/or hyperphosphatemia and PTH values between 500 and 800 pg/mL^6^. The objective of this document was to develop a cost-effectiveness and budgetary impact analysis of cinacalcet versus paricalcitol for patients undergoing dialysis with SHPT, from the perspective of the SUS, after analyzing new scientific evidence on the use of cinacalcet, with a view to expanding its use to treatment of SHPT associated with stage 5D CKD as first-line treatment for patients with PTH > 300 pg/mL in the presence of hyperphosphatemia and/or hypercalcemia, or replacing paricalcitol in patients who have adverse effects of hypercalcemia and/or hyperphosphatemia without improvement after adjusting the dialysis bath, the phosphorus binder and the reduction of the paricalcitol dose or even in association with paricalcitol in those patients who did not reach the target levels of PTH (< 300 pg/mL), as part of the recently published reports and updated “Clinical protocol and therapeutic guidelines for CKD bone and mineral metabolism disorders” and “Cinacalcet for the treatment of patients with hyperparathyroidism secondary to stage 5D chronic kidney disease”^7, 8^.

## Methodology

We searched for evidence in The Cochrane Library, MedLine (via PubMed), Embase (Elsevier), PubMed Central, Epistemonikos, NICE and Virtual Health Library databases. Finally, the review Palmer et al.^9^, published in 2020, was included for evidence synthesis. Regarding the primary outcomes, there was a statistically significant difference between the group receiving cinacalcet compared to the control group for PTH levels (SMD = –1.78; 95%CI: –2.75, –0.82; p < 0.00001); but there were no significant differences for all-cause mortality (RR = 0.96; 95%CI: 0.62–1.50; p = 0.87) and cardiovascular mortality (RR = 0.25; 95%CI: 0.03–2.28; p = 0.22). For secondary endpoints, there was a statistically significant difference between the group receiving cinacalcet compared to the control group for serum calcium levels (SMD = –4.90; 95%CI: –6.75, –3.04; p < 0.00001), serum phosphorus levels (SMD = –1.19; CI95%: –2.01, –0.37; p < 0.00001) and Ca x P product (SMD = –3.00; CI95%: –5.49, –0.50; p < 0.00001). The use of cinacalcet was also statistically significant in preventing parathyroidectomy when compared to the standard treatment (RR = 0.21; 95% CI: 0.05–0.83; p < 0.03). There was no significant difference between groups for reduction in the incidence of cardiac events (RR = 1.62; 95%CI: 0.61–1.43; p = 0.33) and in the prevention of fractures (RR = 0.52; 95%CI: 0.12–2.27; p-value = 0.39). Regarding technology safety outcomes, an increased risk for gastrointestinal events such as nausea (RR = 2.39; CI: 1.23–4.66; p < 0.01) was observed for the group that received cinacalcet. An increased risk in the incidence of hypocalcemia was also observed in the group receiving cinacalcet compared to the control group (RR = 8.46; CI: 5.48–13.05; p < 0.00001). According to GRADE, the quality of evidence was rated as moderate for mortality, parathyroidectomy, and most safety outcomes. In general, the others were of low quality of evidence.

## Economic Evaluation

**Chart 1:**
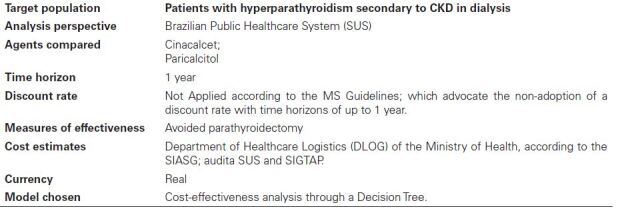
Characteristics of the cost-effectiveness analysis model

Based on literature data, an economic evaluation was performed to estimate the incremental cost-effectiveness ratio (ICER) of cinacalcet compared to paricalcitol for the treatment of hyperparathyroidism secondary to stage 5D chronic kidney disease. The study design followed premises of the Methodological Guidelines for Economic Evaluation of the Ministry of Health^10^. In order to increase the transparency of the proposed study, the main aspects of the studies were summarized according to the CHEERS Task Force Report^11^ checklist (Chart 1).

## Estimate of Resources and Costs

For paricalcitol, using a 1:5 ratio of calcitriol to paricalcitol, it would be 5 mcg/every other day of paricalcitol (15 mcg per week divided into 3 dialysis sessions). For cinacalcet, a dose of 90 mg per day was considered (90 pills of 30 mg per month). The value of the drugs paricalcitol and cinacalcet considered for the calculation of treatment costs was the weighted average of purchases made in the last 18 months by the Health Logistics Department (DLOG) of the Ministry of Health, according to SIASG, via the Health Price Bank (BPS) (accessed on Nov. 18, 2021). Other direct costs, such as consultations and laboratory tests, were not considered.

**Chart 2:**
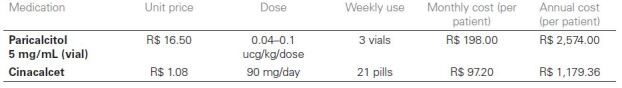
Monthly and annual mean costs of paricalcitol and cinacalcet per patient

Chart 2 shows the average monthly and annual cost of paricalcitol and cinacalcet per patient.

## Efficiency

The transition probabilities between states (hospitalization for parathyroidectomy) were obtained from the literature (PubMed). The probability of parathyroidectomy was extracted from the SR by Palmer et al.^9^, published in 2020, and data from the SBN, being 10% in the group using paricalcitol and 2.1% in the group using cinacalcet (RR 0, 31).

## Economic Model

**Figure 1.**
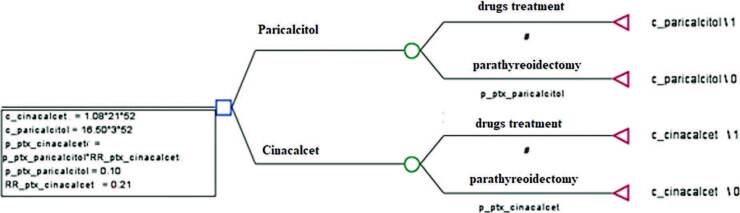
Decision tree for the cost-effectiveness analysis.

The analytical model adopted was the decision tree for conducting the economic evaluation in the TreeAge Pro 2009 software^12^. Two possibilities were considered in the model: continuing to use the medication (dialysis) and performing a parathyroidectomy (ptx). The format of the decision tree is shown below (Figure 1).

## Budgetary Impact Analysis

An analysis was carried out to estimate the budgetary impact of expanding the use of cinacalcet, in the SUS, for the treatment of SHPT to CKD in dialysis patients.

The analysis of the budgetary impact adopted the perspective of the Brazilian public healthcare system (SUS), as it is the holder of the budget at the federal level, as recommended by the Methodological Guideline for the Analysis of Budgetary Impact of the Ministry of Health (MS)^13^.

The time horizon adopted was five years, according to MS Guidelines.

## Proposed Price for Incorporation

In consultation with the Health Price Bank (BPS), the most recent purchases of cinacalcet hydrochloride were identified, in the presentations of 30 mg and 60 mg tablets, by the Health Logistics Department of the Ministry of Health (DLOG/MS) in amount of R$ 1.08 and R$ 2.17, respectively; in the period from 04/18/2020 to 10/18/2021. In the same period, a purchase made by the DLOG of paricalcitol was also identified, in the amount of R$ 16.50 per unit. For calcitriol, the value of R$ 1.09 was used relative to the weighted average of the price practiced in public purchases carried out in the last 18 months, according to the SIASG, since purchases made by the DLOG/MS^12^ were not identified (Chart 2).

## Treatment Costs

For oral calcitriol, a dose of 1 mcg was considered on alternate days (3 mcg/week divided into 3 dialysis sessions) and for injectable paricalcitol, it would be 5 mcg on alternate days (15 mcg/week divided into 3 dialysis sessions), using a 1:5 proportion of calcitriol in relation to paricalcitol. For cinacalcet, a dose of 90 mg per day was considered (90 pills of 30 mg per month). To estimate drug costs, the value of R$ 16.50 was used for the unit of paricalcitol, considering the identification of a purchase made by DLOG/MS, and for calcitriol the weighted average was used (R$ 1.09) of the price practiced in public purchases carried out in the last 18 months, both verified in the BPS. Other direct costs, such as consultations and laboratory tests, were not considered.

Chart 2 shows the average monthly and annual cost of cinacalcet and paricalcitol, per patient.

## Population

**Chart 3.**
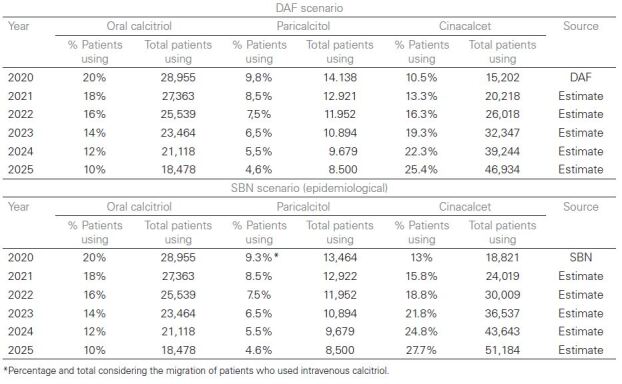
Population estimates in both scenarios considered

Three scenarios were considered: the main one of measured demand, based on data from the Department of Pharmaceutical Assistance and Strategic Inputs of the Ministry of Health (DAF); the alternative of measured demand, based on data from the Open Room on Health Intelligence (SABEIS)^10^; and the epidemiological alternative, based on data from the Brazilian Society of Nephrology (SBN), according to Chart 3.

According to the main scenario, DAF data show that, in 2020, 15,202 patients (10.5% of the dialysis population) used cinacalcet and 14,138, paricalcitol (9.8% of the dialysis population). With increased use, it is estimated that half of the patients using vitamin D analogues would have indication of cinacalcet for presenting PTH above 500 pg/mL in the presence of hyperphosphatemia or hypercalcemia or for not reaching the PTH target value, between 150–300 pg/mL with the use of at least 0.1 ucg/kg/dose of paricalcitol or 3 ucg/week of calcitriol or even because of having a kidney transplant with PTH > 120 pg/mL, increasing from 10.5% to 25.4% of patients on dialysis using cinacalcet in 5 years^4^.

According to the alternative scenario of epidemiological approach, the prevalent dialysis population of 144,779 patients was considered, according to the SBN Dialysis Census, 2020, with annual growth of the dialysis population of 5%. Of these, around 18% of patients had moderate SHPT (PTH above 600 pg/mL) which totals 26,060 patients with a potential indication for the use of cinacalcet, as long as the patient does not have hypocalcemia. According to SBN epidemiological data, around 13% of patients were using cinacalcet, 4.9% of patients were using paricalcitol, 4.4% were using intravenous calcitriol and 20% were using oral calcitriol in 2020. In the scenario without incorporation, the proportions of use of cinacalcet were maintained at 13% and oral calcitriol at 20%. Due to the discontinuity of intravenous calcitriol, we considered the migration of these patients to paricalcitol, resulting in a proportion of use of 9.3%^4^.

With the expansion of use, it is estimated that half of the patients who use vitamin D analogues would be indicated for cinacalcet because they have PTH above 500 pg/mL in the presence of hyperphosphatemia or hypercalcemia or because they do not reach the PTH target value, between 150–300 pg/mL with the use of at least 0.1 ucg/kg/dose of paricalcitol 3 times a week or even for having a kidney transplant with PTH > 120 pg/mL. Therefore, in the scenario with increased use of cinacalcet, considering a gradual increase over 5 years, the population on dialysis using cinacalcet would increase from 13% to 27.7%.

## Results

### Cost-Effectiveness Evaluation

The analysis showed that the use of cinacalcet results in one-off savings of R$ 1,394.64 per year and an incremental effectiveness of 0.08, in relation to avoided parathyroidectomy. The ICER was – R$ 17,653.67 per parathyroidectomy avoided for cinacalcet, as it proved to be more effective and cheaper compared to paricalcitol. Therefore, cinacalcet was dominant in avoiding parathyroidectomies.

### Budgetary Impact Analysis

#### Main Scenario – DAF Data (Measured Demand)

In the main scenario considering DAF data for measured demand, an incremental budgetary impact was estimated with the expansion of the use of cinacalcet of - R$ 1,640,864.62 in the first year, and - R$ 10,740,743.86 at the end of five years (Table 1), that is, representing savings of resources for the SUS.

**Table 1:** Budgetary impact in 5 years for the treatment of SHPT secondary to CKD in the dialysis population using the Vitamin D analogues with the expansion of cinacalcet use (DAF and epidemiological scenario)

DAF scenario					
Year	Eligible population	Budgetary impact with oral calcitriol* or paricalcitol** (baseline scenario)	Diffusion rate for cinacalcet	Budgetary impact with cinacalcet*** and calcitriol* or paricalcitol** (proposed scenario)	Incremental budgetary impact with cinacalcet
2021	40,284	R$ 73,103,631.98	13.3%	R$ 71,462,767.36	- R$ 1,640,864.62
2022	37,491	R$ 76,759,342.56	16.3%	R$ 74,826,280.51	- R$ 1,933,062.05
2023	34,358	R$ 80,597,309.69	19.3%	R$ 78,345,556.73	- R$ 2,251,752.96
2024	30,797	R$ 84,627,151.13	22.3%	R$ 82,029,671.57	- R$ 2,597,479.56
2025	26,978	R$ 88,858,484.64	25.4%	R$ 86,540,899.97	- R$ 2,317,584.67
**Total in 5 years**		**R$ 403,945,920.00**		**R$ 393,205,176.14**	**- R$ 10,740,743.86**

Epidemiological scenario					
Year	Eligible population	Budgetary impact with oral calcitriol* or paricalcitol** (baseline scenario)	Cinacalcet diffusion rate	Budgetary impact with cinacalcet*** and calcitriol* or paricalcitol** (proposed scenario)	Incremental budgetary impact with cinacalcet
2021	40,284	R$ 75,730,503.02	15.8%	R$ 75,896,871.52	R$ 166,368.50
2022	43,297	R$ 79,517,576.16	18.8%	R$ 79,480,799.71	- R$ 36,776.45
2023	40,056	R$ 83,493,454.97	21.8%	R$ 83,232,801.89	- R$ 260,656.08
2024	36,076	R$ 87,668,102.81	24.8%	R$ 87,161,277.53	- R$ 506,825.8
2025	31,043	R$ 92,051,483.04	27.7%	R$ 91,498029.98	- R$ 553,453.06
**Total in 5 years**		**R$ 418,461,120.00**		**R$ 417,269,780.63**	**- R$ 1,191,339.37**

* Annual cost with oral calcitriol treatment, per patient = R$ 627.84;

** Annual cost with paricalcitol treatment per patient = R$ 2,574.00;

*** Annual cost of treatment of cinacalcet, per patient = R$1,179.36.

#### Alternative Scenario – SBN Data (Epidemiological)

Table 1 shows the budgetary impact of the epidemiological scenario without expanding the use and with expanding the use of cinacalcet in 1 to 5 years, with the incremental impact being BRL 166,368.50 in the first year, and - BRL 1,191,339.37 at the end of five years, that is, representing savings for the SUS.

The incremental budgetary impact with the expansion of the use of cinacalcet in the SUS will be between - R$ 1,640,864.62 and R$ 166,368.50 in the first year, considering the main scenario, based on DAF data, and the epidemiological scenario, based on in the SBN data. At the end of 5 years after the expansion of use, an incremental impact was estimated between - R$ 10,740,743.86 and - R$ 1,191,339.37; considering the same scenarios.

## Discussion

In this study, patients with SHPT at CKD stage 5 were evaluated in order to perform a cost-effectiveness analysis and budgetary impact of cinacalcet versus paricalcitol, from the perspective of the SUS. It was decided not to develop a Markov decision model^12^, because the chosen time horizon was one year.

The systematic review by Palmer et al.^9^, published in 2020 and included for the synthesis of evidence, showed, in relation to the primary outcomes, that there was a statistically significant difference between the group that received cinacalcet compared to the control group for levels of PTH, but no significant differences were observed for all-cause mortality and cardiovascular mortality. For secondary endpoints, there was a statistically significant difference between the group receiving cinacalcet compared to the control group for serum calcium levels, serum phosphorus levels, and Ca x P product. The use of cinacalcet was also statistically significant in preventing parathyroidectomy, when compared to standard treatment (RR = 0.21; 95%CI: 0.05–0.83; p < 0.03). There was no significant difference between groups for reducing the incidence of cardiac events and preventing fractures. Regarding the technology’s safety outcomes, there was an increased risk for gastrointestinal events such as nausea and hypocalcemia for the group that received cinacalcet. According to GRADE, the quality of evidence was rated as moderate for mortality, parathyroidectomy, and most safety outcomes. In general, the others were of low quality of evidence.

Although without additional benefits in terms of mortality in dialysis patients, cinacalcet has superior efficacy and safety similar to paricalcitol, reducing the risk of parathyroidectomy in dialysis patients, which is a complex surgery and only performed in certain referral services. In view of the evidence, for the cost-effectiveness analysis, the outcome parathyroidectomy avoided was considered. As a result of comparing cinacalcet versus vitamin D analogues (paricalcitol) from the SUS perspective, the cost-effectiveness analysis showed that the use of cinacalcet results in one-off savings of BRL 1,394.64 per year and an incremental effectiveness of 0.08, in relation to avoided parathyroidectomy. The incremental cost-effectiveness ratio (ICER) was - BRL 17,653.67 per parathyroidectomy avoided for cinacalcet, as it proved to be more effective and cheaper compared to paricalcitol.

As for the BIA, it was estimated that the incremental budgetary impact with the expansion of the use of cinacalcet in the SUS will be between - R$ 1,640,864.62 and R$ 166,368.50 in the first year, considering the main scenarios based on DAF data and SABEIS and the epidemiological scenario based on SBN data. At the end of 5 years after the expansion of use, an incremental impact was estimated between - R$ 10,740,743.86 and - R$ 1,191,339.37; considering the same scenarios.

The main limitation of the present study concerns the estimation of the target population, which was estimated based on data from SBN records. Although there are epidemiological data on the population on dialysis, with SHPT at CKD and with levels of PTH, calcium and phosphorus above the target, these are estimated data, based on records, which may be underestimated, considering that 40% of Brazilian centers of dialysis participated in the 2020 Census, most of them being academic. This hypothesis is strengthened when we compare the epidemiological data from the SBN with the SABEIS acquisition records, which are 40% higher than the data reported by the SBN. Another limitation pointed out is that it was not possible to estimate the economic impact of cinacalcet among patients on peritoneal dialysis, separately.

Another point to be highlighted is that the predicted diffusion rate in the three scenarios was defined through assumptions related to the future use of cinacalcet in the SUS, which is still very uncertain. Finally, another limitation of the BIA is not knowing the number of patients with contraindications to the use of cinacalcet and not obtaining the number of patients using calcitriol by DAF or SABEIS, since the drug is also dispensed for other ICDs.

## Conclusion

The results presented in this study show that, from the SUS perspective, the treatment of patients with SHPT on dialysis with cinacalcet is cost-effective, compared to paricalcitol, with an ICER of - R$ 17,653.67 per parathyroidectomy avoided. As for the BIA, it was estimated that the incremental budgetary impact with the expansion of the use of cinacalcet in the SUS will be between - R$ 1,640,864.62 and R$ 12,754,246.38 in the first year, considering the main scenario, based on the DAF and SABEIS data, and the epidemiological scenario, based on SBN data. At the end of 5 years after the expansion of use, an incremental impact was estimated between - R$ 10,740,743.86 and R$ 94,812,141.73; considering the same scenarios. Therefore, cinacalcet was dominant in avoiding parathyroidectomies, being cost-effective.
